# Conventional versus minimally invasive extra-corporeal circulation in patients undergoing cardiac surgery: A randomized controlled trial (COMICS)

**DOI:** 10.1177/02676591241258054

**Published:** 2024-06-04

**Authors:** Gianni D Angelini, Barnaby C Reeves, Lucy A Culliford, Rachel Maishman, Chris A Rogers, Kyriakos Anastasiadis, Polychronis Antonitsis, Helena Argiriadou, Thierry Carrel, Dorothée Keller, Andreas Liebold, Fatma Ashkaniani, Aschraf El-Essawi, Ingo Breitenbach, Clinton Lloyd, Mark Bennett, Alex Cale, Serdar Gunaydin, Eren Gunertem, Farouk Oueida, Ibrahim M Yassin, Cyril Serrick, John M Murkin, Vivek Rao, Marco Moscarelli, Ignazzo Condello, Prakash Punjabi, Cha Rajakaruna, Apostolos Deliopoulos, Daniel Bone, William Lansdown, Narain Moorjani, Sarah Dennis

**Affiliations:** 1 1980 Bristol Medical School, University of Bristol, Bristol, UK; 2 37783 Aristotle University of Thessaloniki School of Medicine, Thessaloniki, Greece; 3 27252 University Hospital Bern, Bern, Switzerland; 4 27197 Universitätsklinikum Ulm, Ulm, Germany; 5 Universitätsmedizin Göttingen, Göttingen, Germany; 6 Klinikum Braunschweig, Braunschweig, Germany; 7 University Hospitals Plymouth NHS Trust, Plymouth, UK; 8 4020 Hull University Teaching Hospitals NHS Trust, Hull, UK; 9 Numune Training and Research Hospital in Ankara, Ankara, Turkey; 10 289167 Saud Al-Babtain Cardiac Centre, Dammam, Saudi Arabia; 11 7989 University Health Network, Toronto, ON, Canada; 12 6221 University of Western Ontario, London, ON, Canada; 13 46804 Anthea Hospital Bari, Italy; 14 156647 Imperial College Healthcare, London, UK; 15 1984 University Hospitals Bristol NHS Foundation Trust, Bristol, UK; 16 AHEPA University Hospital, Thessaloniki, Greece; 17 2144 Royal Papworth Hospital, Cambridge, UK

**Keywords:** Coronary artery bypass grafting, aortic valve replacement, extracorporeal circulation, cardiopulmonary bypass, randomized controlled trial

## Abstract

**Introduction:**

The trial hypothesized that minimally invasive extra-corporeal circulation (MiECC) reduces the risk of serious adverse events (SAEs) after cardiac surgery operations requiring extra-corporeal circulation without circulatory arrest.

**Methods:**

This is a multicentre, international randomized controlled trial across fourteen cardiac surgery centres including patients aged ≥18 and <85 years undergoing elective or urgent isolated coronary artery bypass grafting (CABG), isolated aortic valve replacement (AVR) surgery, or CABG + AVR surgery. Participants were randomized to MiECC or conventional extra-corporeal circulation (CECC), stratified by centre and operation. The primary outcome was a composite of 12 post-operative SAEs up to 30 days after surgery, the risk of which MiECC was hypothesized to reduce. Secondary outcomes comprised: other SAEs; all-cause mortality; transfusion of blood products; time to discharge from intensive care and hospital; health-related quality-of-life. Analyses were performed on a modified intention-to-treat basis.

**Results:**

The trial terminated early due to the COVID-19 pandemic; 1071 participants (896 isolated CABG, 97 isolated AVR, 69 CABG + AVR) with median age 66 years and median EuroSCORE II 1.24 were randomized (535 to MiECC, 536 to CECC). Twenty-six participants withdrew after randomization, 22 before and four after intervention. Fifty of 517 (9.7%) randomized to MiECC and 69/522 (13.2%) randomized to CECC group experienced the primary outcome (risk ratio = 0.732, 95% confidence interval (95% CI) = 0.556 to 0.962, *p* = 0.025). The risk of any SAE not contributing to the primary outcome was similarly reduced (risk ratio = 0.791, 95% CI 0.530 to 1.179, *p* = 0.250).

**Conclusions:**

MiECC reduces the relative risk of primary outcome events by about 25%. The risk of other SAEs was similarly reduced. Because the trial terminated early without achieving the target sample size, these potential benefits of MiECC are uncertain.

## Introduction

Contemporary cardiac surgery incorporates advances in surgical technique as well as cardiopulmonary bypass (CPB) technology aiming to minimize the invasiveness of surgery in a high-risk and aging population, often with many comorbidities.^1^ Despite decreasing mortality, patients having cardiac surgery continue to experience significant ‘real world’ postoperative morbidity.^2^ This is mainly attributed to the unavoidable consequences of CPB triggered by contact of blood with artificial surfaces, activation of coagulation, haemodilution and hypoperfusion resulting in microcirculatory derangement.^3^ The concept of “more physiologic” intraoperative perfusion has evolved to address these harms of surgery, with minimally invasive extracorporeal circulation (MiECC) as a core element. MiECC systems have been developed using the concept of a closed circuit to preserve microcirculation and enhance organ perfusion, while preserving coagulation integrity.^4^

Several meta-analyses,^5–8^ including a large network meta-analysis which compared MiECC to off-pump coronary artery bypass as well as conventional extracorporeal circulation (CECC),^9^ concluded that MiECC has substantial benefits over CECC, reducing the risk of death and in-hospital postoperative complications by about 50%. However, included Randomised Controlled Trials (RCTs) were mostly small and of moderate quality and MiECC has not been widely adopted, despite its apparent promise.

We aimed to carry out a large multicentre trial to test the hypothesis that, compared to CECC, MiECC reduces the proportion of patients experiencing postoperative morbidity. The trial was designed to address most limitations of previous trials by evaluating MiECC systems that met specified criteria, being large enough to influence clinical practice and with features to prevent bias.

## Methods

### Trial design

COMICS was a pragmatic, multicentre, two-group parallel RCT to evaluate MiECC in all patients having elective or urgent coronary artery bypass grafting (CABG), aortic valve replacement (AVR) or CABG + AVR using extra-corporeal circulation without circulatory arrest. The study was designed with two stages: stage 1 was an internal pilot trial to determine the feasibility of the trial and stage 2 was the main trial. Full details of the study rationale and design are reported elsewhere.^10^ The South-West Central Bristol Research ethics committee approved the trial protocol on 29 Nov 2017 (UK Integrated Research Application System reference 222991). The trial was registered as ISRCTN92590475 before recruitment began.

### Participants and setting

The setting was cardiac surgery centres in the UK, Europe, Turkey, Saudi Arabia and Canada. We planned to use eligibility criteria that were as inclusive as possible, to promote the applicability of the trial findings. The reference population was all patients aged ≥18 and <85 years, having elective or urgent cardiac surgery for CABG only, AVR only, or CABG + AVR surgery, using extra-corporeal circulation without circulatory arrest. A patient was ineligible if any of the following applied: requirement for major aortic surgery (e.g. aortic root replacement), contraindication or objection to transfusion of blood products, congenital or acquired platelet, red cell or clotting disorders (patients with iron deficient anaemia were not excluded), unable to give informed consent for the study.

A centre had to agree to randomize participants eligible for at least one of the operation strata and be familiar with and using MiECC for usual care for operations of the type for which participants were being recruited.

### Interventions

MiECC systems have evolved to address safety, volume and blood management issues and have been classified according to their features (Types I, II, III and IV).^11^ Centres were allowed to use any MiECC circuit which used CE-marked components (or components which conform to the required standards for countries outside the European Community) and which had features consistent with a Type II, III or IV system.

CECC had to comprise (required components): a standard oxygenator, roller pump, hard-shell reservoir, arterial filter, shed-blood suctions, any of a range of venting options, uncoated tubing, and a cell-saver device. The following optional/alternative components could be integrated: a coated oxygenator, coated tubing and centrifugal pump. Use of a soft-shell reservoir or vacuum assisted venous drainage were prohibited because these components made CECC resemble a custom-made MiECC circuit.

Given the pragmatic design of the trial, other aspects of the operations (e.g. cardioplegia solution and infusion mode, patient’s body temperature) were allowed to vary by operation and centre but had to be consistent in the MiECC and CECC groups. We collected operative details to characterize and report these variations.

### Outcomes

The primary outcome was a composite of post-operative serious adverse events (SAEs) up to 30 days after surgery following the index admission. Any of the following 12 events classified a participant as having the primary outcome: death, myocardial infarction (MI; suspected events were documented by serum troponin concentrations and electrocardiograph recording (ECG) but the latter were not provided), stroke (report of brain imaging by CT or MRI, in association with new onset focal or generalized neurological deficit), gut infarction (diagnosed by laparotomy or post mortem), stage 3 AKI or need for haemofiltration,^12,13^ reintubation, tracheostomy, mechanical ventilation >48 h, reoperation, percutaneous intervention, sternal wound infection with dehiscence, septicaemia confirmed by microbiology. We intended that SAEs qualifying for the primary outcome were objectively defined (above) or confirmed by documentation.

Secondary outcomes (collected by centres up to 30 days after surgery) were: all-cause mortality, any SAE not included in the primary outcome, units of red blood cells (RBCs) transfused, units of other blood products (fresh frozen plasma (FFP), platelets or cryoprecipitate), time to discharge from cardiac ICU after surgery, time to discharge from hospital following surgery, delirium in ICU, assessed with the Intensive Care Delirium Screening Checklist^13^ for up to 5 days. Health-related quality-of-life (HRQoL) was reported by participants using the EuroQol (EQ-5D-5L^14^; both index score and visual analogue score) at 30 and 90 days after surgery. Participants also reported health and social care resources and associated costs up to 90 days after surgery at 90 days. Delirium was only assessed by centres with the capability. An economic evaluation alongside the trial using quality-adjusted life years based on the EQ-5D-5L as the primary outcome was planned.

### Sample size

The trial was designed to test the hypothesis that MiECC reduces the proportion of patients experiencing the primary outcome compared to CECC. Based on data from a previous trial,^15^ the proposed composite outcome was expected to occur in 15% to 18% of patients having surgery with CECC, the percentage depending on the proportions in the three surgical strata (an overall frequency of 15% assumed 70% of participants in the CABG stratum, 20% in the AVR stratum, 10% in the CABG + AVR stratum; 18% assumed 40%, 40% and 20% respectively). For illustration, to detect a risk ratio of ≤0.75 with 90% power and 5% significance (2-tailed), a sample size of 2,504 (20% vs 15%) to 3,258 (16% vs 12%) was required. We planned to recruit 3,500 patients to allow for uncertainty in the assumptions underpinning the calculation.^16–18^

### Randomization

Randomization was stratified by centre and surgical stratum; the latter was considered an important prognostic variable because it influences duration of extracorporeal circulation. Randomization occurred as close to surgery as possible, using a secure internet-based randomization system (Sealed Envelope^TM^, https://www.sealedenvelope.com) to ensure allocation concealment. Randomized allocations were generated in advance by Sealed Envelope using random permuted blocks of sizes 4, 6 and 8. Information to identify a participant and to confirm eligibility had to be entered before allocation was assigned. Randomisation was performed by a designated member of the local research team.

### Features to minimize bias

Participants were not told their allocations. Documentary evidence was sought wherever possible for primary outcome events. The 30-days primary outcome minimised loss to follow-up. Reporting bias was minimised by a statistical analysis plan (SAP) finalised before the database was locked.

### Statistical methods

Analyses were based on a pre-specified SAP and performed on an intention-to-treat basis. The primary outcome was compared using modified Poisson regression. All models were adjusted for stratification factors with operation type fitted as a fixed effect and centre as a random effect. Centres with fewer than 10 randomized participants were grouped together into a single centre. The primary analysis did not adjust for any other baseline variable. A significance threshold of 0.05 was used for all analyses. The treatment effect for binary outcomes was specified as the risk ratio (RR) of MiECC versus CECC, reported as an adjusted RR with 95% confidence interval (CI) and *p*-value. In secondary analyses, risk differences and 95% CIs were estimated by binomial regression and odds ratios and 95% CIs were estimated by logistic regression. Six subgroup analyses were specified: age, sex, operation (CABG, AVR, CABG + AVR), EuroSCORE II dichotomized at the median, preoperative renal function (normal vs moderate, severe renal impairment or hemofiltration), and preoperative haemoglobin dichotomized at the median.

Time to event outcomes were analysed using Cox proportional hazards models with a shared frailty term of centre. Continuous longitudinal outcomes were analysed using repeated measures mixed effects models. Treatment effects were specified as hazard ratios (HRs) or mean differences (MDs), after data transformation if necessary. In the event, distributions of units of RBCs transfused, other blood products transfused and EQ-5D-5L index score could not be modelled as continuous variables because a large proportion of participants had values of zero or one; the data were dichotomized (any RBC transfusion; any other blood product transfusion; EQ-5D-5L index score <1). Other data were summarized descriptively.

### Governance and funding sources

The University of Bristol was the lead coordinating centre and UK sites participated under the UK ethics approval. Non-UK sites were responsible for their own local governance processes and obtaining the necessary approvals. Independent trial steering and data monitoring and safety committees (TSC and DMSC) were established and reviewed accruing data. The trial was funded by the British Heart Foundation, the UK National Institute for Health Research Bristol Biomedical Research Centre at University Hospitals Bristol NHS Foundation Trust and the University of Bristol and unconditional charitable grants from Medtronic and Maquet. The funders had no role in the design or conduct of the trial or in data analyses or reporting.

## Results

### Recruitment

A total of 1084 patients at 14 recruiting centres gave written informed consent to take part, of whom 1071 were randomized between 8 May 2018 and 20 December 2021, 536 to CECC and 535 to MiECC. Recruitment to the trial was stopped early on the recommendation of the Trial Steering Committee (TSC). Recruitment had been slow during the COVID-19 pandemic and had decreased in the second half of 2021 due to a new wave of infections.

**Figure 1.**
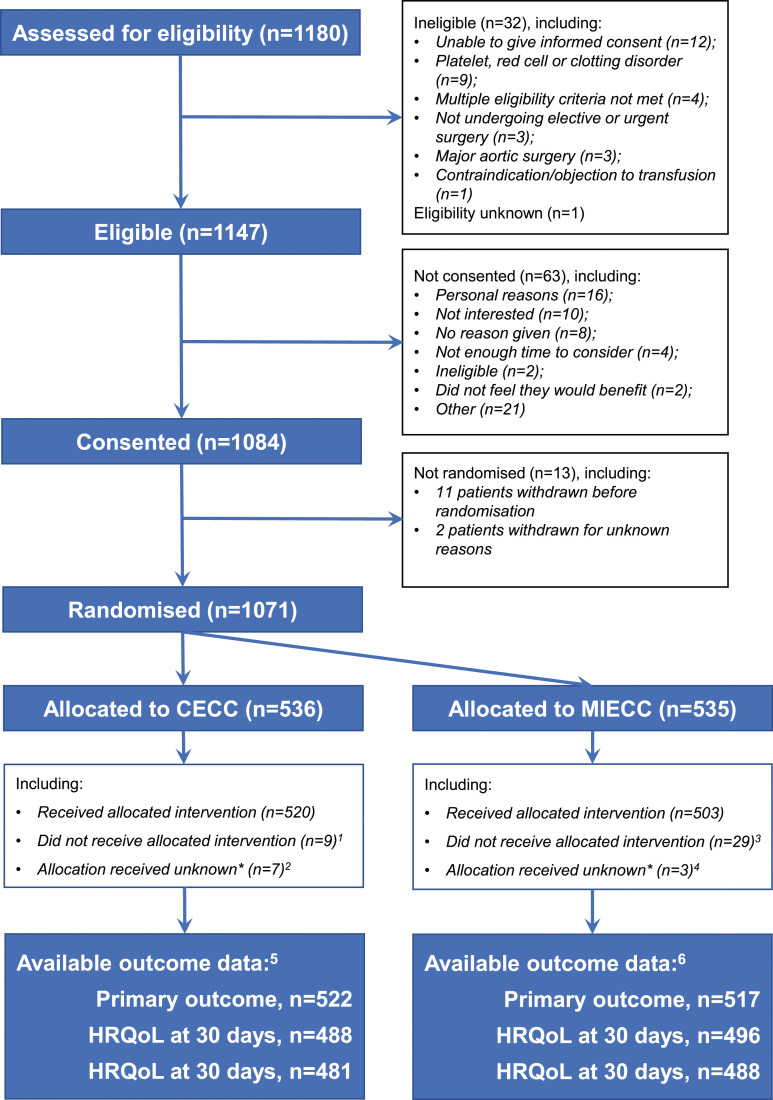
Flow of patients in the trial. CECC – conventional extra-corporeal circulation; HR – hazard ratio; MiECC – minimally invasive extra-corporeal circulation; RBC – red blood cell; RR – risk ratio; SAE – serious adverse event. ^1 ^2 patients received MiECC and 7 patients received either MiECC nor CECC (5 withdrew before the operation). ^2^ 6 patients withdrew. ^3^ 18 patients received CECC and 11 patients received neither MiECC nor CECC (5 withdrew before the operation). ^4^ three patients withdrew. ^5^ 10 withdrawals before 30 days; 2 lost to follow-up; 2 missing data for one or more primary outcome events ^6^ 11 withdrawals before 30 days; 6 lost to follow-up; 1 missing data for one or more primary outcome events.

The flow of participants in the trial is shown in Figure 1. 14 patients in the CECC group and 18 patients in the MiECC group could not be included in the primary outcome analysis; 21 withdrew, 8 were lost to follow-up and three had missing primary outcome data. The remaining 1039 (97%) comprised 522 allocated to CECC and 517 allocated to MiECC. Protocol deviations and withdrawals are summarized in the supplement (Supplemental Tables 1 and 2). In the analysis of the primary outcome, three patients in the CECC group and 17 in the MiECC group did not receive their allocated intervention (2 patients in the CECC group received MiECC, 1 received neither MiECC or CECC; 15 patients in the MiECC group received CECC, 2 received neither MiECC nor CECC).

### Baseline data

Patient characteristics, pre-operative medications, operations (stratified), operative priority pre-operative details were distributed similarly in the two groups (see Table 1 and Supplemental Table 3). The median age was 66 years and 83% (873/1051) of participants were male. The majority had a left ventricular ejection fraction >50% (71%, 741/1051), CCS class <III (79%, 790/1002) and NYHA class I or II (78%, 820/1051). The median EuroSCORE II was 1.24 (interquartile range, IQR, 0.83, 2.05).

**Table 1.** Patient demography, medical history, pre-operative medications and operations.

	Randomised to CECC (*n* = 536)		Randomised to MiECC (*n* = 535)		Overall (*n* = 1071)	
	*n*	%	*n*	%	*n*	%
Demography						
Male gender	440/525	83.8%	433/526	82.3%	873/1051	83.1%
Age (years, median, IQR)^a^	67.0	(58.0, 72.0)	66.0	(60.0, 73.0)	66.0	(59.0, 73.0)
BMI (median, IQR)^a^	28.10	(25.5, 31.0)	28.0	(25.3, 30.9)	28.1	(25.4, 31.0)
Medical history						
Renal impairment						
Normal (CC >85 mL/min)	320/525	61.0%	324/526	61.6%	644/1051	61.3%
Moderate (CC >50 & <85)	178/525	33.9%	176/526	33.5%	354/1051	33.7%
Severe (CC <50)	24/525	4.6%	21/526	4.0%	45/1051	4.3%
Dialysis (regardless of CC)	3/525	0.6%	5/526	1.0%	8/1051	0.8%
Extracardiac arteriopathy	97/525	18.5%	96/526	18.3%	193/1051	18.4%
Active endocarditis	3/525	0.6%	2/526	0.4%	5/1051	0.5%
Poor mobility	23/525	4.4%	18/526	3.4%	41/1051	3.9%
Critical preoperative state	5/525	1.0%	7/526	1.3%	12/1051	1.1%
Previous cardiac surgery	5/525	1.0%	6/526	1.1%	11/1051	1.0%
Diabetes on insulin	98/525	18.7%	82/526	15.6%	180/1051	17.1%
Chronic pulmonary lung disease	56/525	10.7%	71/526	13.5%	127/1051	12.1%
NYHA						
I	136/525	25.9%	145/526	27.6%	281/1051	26.7%
II	272/525	51.8%	267/526	50.8%	539/1051	51.3%
III	107/525	20.4%	104/526	19.8%	211/1051	20.1%
IV	10/525	1.9%	10/526	1.9%	20/1051	1.9%
LV function						
Good (LVEF >50%)	366/525	69.7%	375/526	71.3%	741/1051	70.5%
Moderate (LVEF 31% - 50%)	145/525	27.6%	138/526	26.2%	283/1051	26.9%
Poor (LVEF 21% - 30%)	12/525	2.3%	12/526	2.3%	24/1051	2.3%
Very poor (LVEF <20%)	2/525	0.4%	1/526	0.2%	3/1051	0.3%
Recent MI	101/525	19.2%	98/526	18.6%	199/1051	18.9%
Pulmonary hypertension						
Moderate (PA systolic 31-55mmHg)	26/523	5.0%	30/526	5.7%	56/1049	5.3%
Severe (PA systolic >55mmHg)	4/523	0.8%	3/526	0.6%	7/1049	0.7%
None	493/523	94.3%	493/526	93.7%	986/1049	94.0%
EuroSCORE II (median, IQR)	1.25	(0.81, 2.08)	1.22	(0.84, 2.04)	1.24	(0.83, 2.05)
Diabetes	223/524	42.6%	188/526	35.7%	411/1050	39.1%
CVA/TIAs	33/524	6.3%	36/526	6.8%	69/1050	6.6%
Atrial fibrillation	25/524	4.8%	32/526	6.1%	57/1050	5.4%
Unstable angina	57/485	11.8%	61/492	12.4%	118/977	12.1%
Congestive heart failure	12/524	2.3%	10/526	1.9%	22/1050	2.1%
Preoperative medications						
Heparin (fractionated/unfractionated)	134/524	25.6%	150/526	28.5%	284/1050	27.0%
Prophylactic	118/134	88.1%	128/150	85.3%	246/284	86.6%
Treatment	16/134	11.9%	22/150	14.7%	38/284	13.4%
Antiplatelet (P2Y12 inhibitors)	129/524	24.6%	131/526	24.9%	260/1050	24.8%
Warfarin	9/524	1.7%	4/526	0.8%	13/1050	1.2%
New oral anticoagulants	32/524	6.1%	31/526	5.9%	63/1050	6.0%
Aspirin not P2Y12	268/368	72.8%	264/360	73.3%	532/728	73.1%
Haemoglobin (mean, SD)^a^	13.88	1.72	13.94	1.64	13.91	1.68
Platelets (mean, SD)^a^	239.88	73.43	235.81	70.17	237.84	71.81
Operative details						
Operation						
CABG	447/530	84.3%	449/532	84.4%	896/1062	84.4%
AVR	49/530	9.2%	48/532	9.0%	97/1062	9.1%
CABG+AVR	34/530	6.4%	35/532	6.6%	69/1062	6.5%
Operative priority						
Emergency	7/524	1.3%	3/523	0.6%	10/1047	1.0%
Elective	459/524	87.6%	453/523	86.6%	912/1047	87.1%
Urgent	58/524	11.1%	67/523	12.8%	125/1047	11.9%

^a^missing for 20 patients (11 randomised to CECC and 9 randomised to MiECC).

CECC - conventional extra-corporeal circulation; MiECC - minimally invasive extra-corporeal circulation; IQR – interquartile range; BMI – body mass index; CC – creatinine clearance; NYHA - New York Heart Association Functional Classification; LV – left ventricular; LVEF – left ventricular ejection fraction; MI – myocardial infarction; PA – pulmonary artery; CVA - cerebrovascular accident; TIA - transient ischaemic attack; SD – standard deviation; CABG – coronary artery bypass graft; AVR – aortic valve replacement.

### Operative details

Most patients had CABG (84%, 896/1092), 9% (97/1062) had AVR and 6% (69/1062) CABG + AVR (type of operation was missing for nine patients). Other operative details such as method of myocardial protection were similar in the two groups (Supplemental Table 4). The mean total cardiopulmonary bypass and cross clamp times were 88 (standard deviation, SD, 30) and 57 (SD 21) minutes respectively. A roller pump was used for 9/519 (1.7%) patients who were allocated to MiECC and a soft-shell reservoir and assisted drainage for 1/522 (0.2%) and 2/521 (0.4%) patients who allocated to CECC. Importantly, other aspects of myocardial protection were similar in the two groups; blood cardioplegia was used for 81% (843/1042) of patients in both groups, warm temperature for 63% and 66% of patients in CECC and MiECC groups, antegrade infusion for 89% of patients in both groups, and intermittent delivery for 99% and 98% of patients in CECC and MiECC group.

### Primary outcome

**Figure 2.**
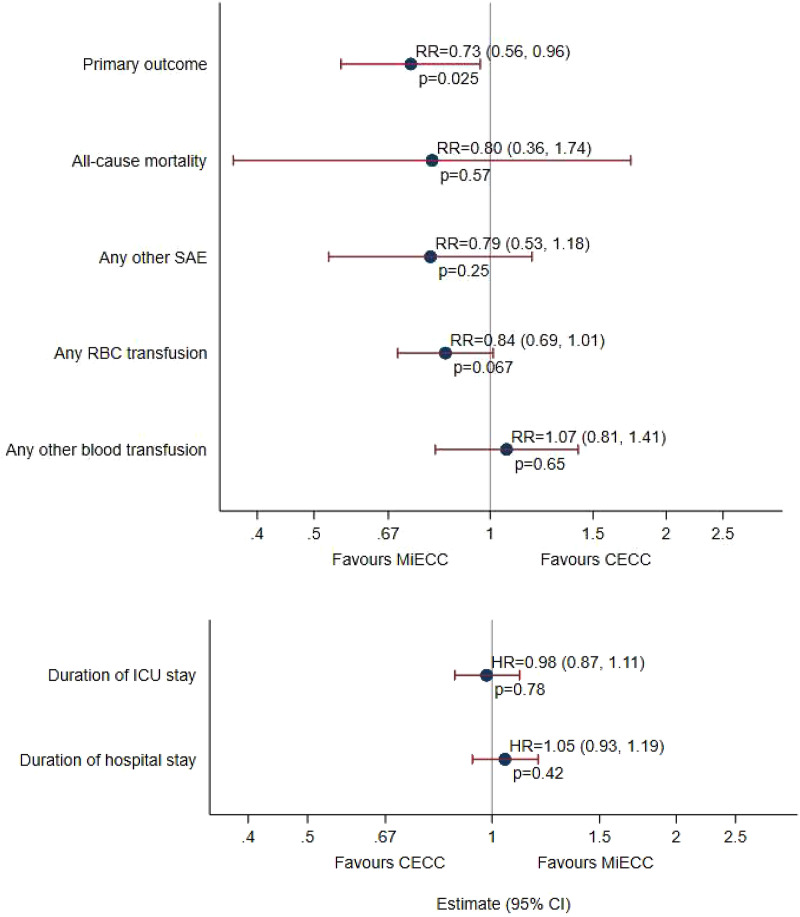
Risk ratios and hazard ratios^1^ for primary and secondary outcomes. CECC – conventional extra-corporeal circulation; HRQoL – health-related quality-of-life; MiECC – minimally invasive extra-corporeal circulation. 1 Hazard ratios >1 represent shorter durations of ICU or hospital stay.

Of 1039 patients with primary outcome data, 69/522 (13.2%) in the CECC group and 50/517 (9.7%) in the MiECC group experienced one or more qualifying events (Supplemental Table 5 and Figure 2). After adjusting for stratification and centre, the RR was 0.73 (95% confidence interval, CI, 0.56 to 0.96, *p* = .025). The risk difference was −3.5% (95% CI -7.0% to 0.3%, *p* = .073). The most frequent qualifying events were reintubation (13 and 26 in the MiECC and CECC groups, overall 39/1044, 3.7%), reoperation (17 and 21 in the MiECC and CECC groups, overall 38/1044, 3.6%), mechanical ventilation >48 h (13 and 14 in the MiECC and CECC groups, overall 27/1037, 2.6%), and stage 3 AKI (13 and 10 in the MiECC and CECC groups, overall 23/1044, 2.2%; frequencies of all qualifying events are described in Supplemental Table 5).

Point estimates for all subgroups lay inside the 95% CI for the primary analysis (Supplemental Figure 1), except for operation type. MiECC reduced the risk for the CABG only and AVR only groups, and increased the risk of the primary outcome for the CABG + AVR group, but not significantly (interaction test, *p* = .48).

### Secondary outcomes

Eight of 522 (1.5%) patients and 10 of 524 (1.9%) died in the MiECC and CECC groups (RR = 0.80, 95% CI 0.36 to 1.74, *p* = .568; Supplemental Table 5 and Figure 2); all but two deaths (both in the MiECC group) occurred in hospital. At least one other SAE not qualifying for the primary outcome occurred in 53/504 (10.5%) and 68/509 (13.4%) patients in the MiECC and CECC groups (RR = 0.79, 95% CI 0.53 to 1.18, *p* = .250; Figure 2).

Due to the distributions, transfusions of blood products were analysed as binary variables, i.e. any versus none, instead of numbers of units. During the operation and hospital stay, RBCs were transfused in 168/518 (32.4%) and 201/521 (38.6%) patients in the MiECC and CECC groups (RR = 0.84, 95% CI 0.70 to 1.01, *p* = .067; Supplemental Table 5). During the same period, any other blood products (FFP, platelets or cryoprecipitate) were transfused in 58/518 (11.2%) and 55/521 (10.6%) patients in the MiECC and CECC groups (RR = 1.07, 95% CI 0.81 to 1.41, *p* = .650; Supplemental Table 5). Units of RBC and other blood products transfused intra- and post-operatively are shown in Supplemental Table 6. Considerably more patients in the CECC group had RBCs transfused intra-operatively, and very slightly more after the operation and before discharge.

Durations of stay in the intensive care unit (24 h) and in hospital after surgery (7 days) did not differ between MiECC and CECC groups (HRs 0.98, 95% CI 0.87 to 1.11, *p* = .78, and 1.05, 95% CI 0.93 to 1.19, *p* = .42 respectively; Supplemental Table 5 and Figure 2).

**Figure 3.**
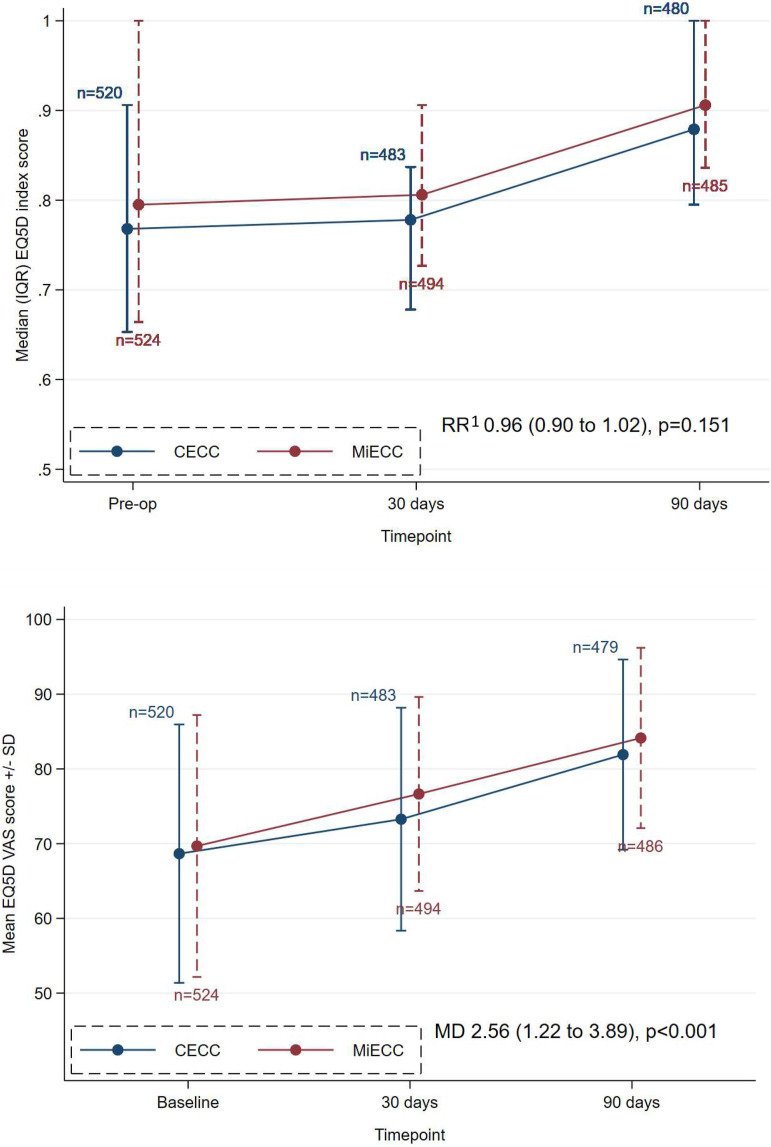
Median EQ-5D-5L index scores (top panel; scale range 0 to 1) and mean EQ-5D-5L visual analogue scores (bottom panel; scale range 0 to 100) in the CECC and MiECC groups at baseline, 30 days and 90 days. CECC – conventional extra-corporeal circulation; EQ-5D-5L – 5-level EuroQol; MD – mean difference; MiECC – minimally invasive extra-corporeal circulation; RR – risk ratio; SD – standard deviation; VAS = visual analogue score. 1 A RR <1 means a non-significantly lower risk of having less than perfect health (index score<1) in the MiECC group than the CECC group.

EQ-5D-5L index and visual analogue scores at either 30 days or 90 days and baseline were available for 92% of participants. There was a small difference in EQ-5D-5L index score at baseline (medians 0.80 and 0.77 in the MiECC and CECC groups), which persisted through 30 days and 90 days. Due to the distribution, index score was analysed as a binary variable, i.e. a score of 1 versus <1. MiECC did not influence the risk of having a score of 1 (RR = 0.96, 95% CI 0.90 to 1.02, *p* = .151; Supplemental Table 5, Figure 3 and Supplemental Table 7). The mean EQ-5D-5L visual analogue score (0 to 100) was distributed more normally, similar in both groups at baseline and analysed as a scaled variable. Mean scores were higher with MiECC (76.6 and 84.1 at 30 and days) than with CECC (73.3 and 81.9) and the overall difference up to 90 days was statistically significantly (MD = 2.56, 95% CI 1.22 to 3.89, *p* < .001; Supplemental Table 5, Figure 3 and Supplemental Table 7).

### Safety

The risk of any SAE not qualifying for the primary outcome, i.e. not hypothesized to be influenced by type of CPB, was a secondary outcome (above and Supplemental Table 5) was reduced by MiECC compared to CECC to a similar extent as the primary outcome, but not to a statistically significantly extent. The frequencies of these SAEs are tabulated in Table 2. SAEs reported as unexpected were few, 43 in 28/515 patients in the CECC group and 43 in 31/515 patients in the MiECC group (Supplemental Table 8) Adverse events in hospital were distributed similarly in the two groups (Supplemental Table 9).

**Table 2.** Frequencies of serious adverse events (SAEs) not qualifying for the primary outcome in CECC and MiECC groups.

	Randomised to CECC (*n* = 536)		Randomised to MiECC (*n* = 535)		Overall (*n* = 1071)	
	*n*/*N*	%	*n*/*N*	%	*n*/*N*	%
Any other SAE (not included in primary outcome)	68/509	13.4	53/504	10.5	121/1013	11.9
Cardiac arrest	7/521	1.3	6/517	1.2	13/1038	1.3
SVT/AF requiring treatment	9/521	1.7	2/517	0.4	11/1038	1.1
VF/VT requiring intervention	1/521	0.2	0/517	0.0	1/1038	0.1
New pacing	2/521	0.4	2/517	0.4	4/1038	0.4
Vasopressors used	6/518	1.2	4/514	0.8	10/1032	1.0
Any inotropes used^a^	4/520	0.8	11/515	2.1	15/1035	1.4
IABP inserted	1/521	0.2	1/517	0.2	2/1038	0.2
Pulmonary artery catheter inserted	0/520	0.0	0/516	0.0	0/1036	0.0
Vasodilator used	8/521	1.5	3/515	0.6	11/1036	1.1
Mask CPAP	4/521	0.8	0/516	0.0	4/1037	0.4
ARDS	2/521	0.4	1/516	0.2	3/1037	0.3
Pneumothorax or pleural effusion requiring drainage	3/521	0.6	4/516	0.8	7/1037	0.7
Peptic ulcer/GI bleed/perforation	0/520	0.0	0/517	0.0	0/1037	0.0
Pancreatitis (amylase >1500iu)	0/520	0.0	0/517	0.0	0/1037	0.0
Ischaemic bowel needing treatment	1/520	0.2	1/517	0.2	2/1037	0.2
Transient ischaemic attack (TIA)	2/520	0.4	0/517	0.0	2/1037	0.2
Deep vein thrombosis (DVT)	0/520	0.0	0/517	0.0	0/1037	0.0
Pulmonary embolus	0/520	0.0	0/517	0.0	0/1037	0.0
Excess bleeding, not needing re-operation	2/520	0.4	5/516	1.0	7/1036	0.7
Pericardial effusion	2/520	0.4	4/517	0.8	6/1037	0.6

^a^1 patient with inotrope use SAE in MiECC group received the alternative treatment.

CECC - conventional extra-corporeal circulation; MiECC - minimally invasive extra-corporeal circulation; SVT - supraventricular tachycardia; AF - atrial fibrillation; VF - ventricular fibrillation; VT - ventricular tachycardia; IABP - intra-aortic balloon pump; CPAP - continuous positive airway pressure; ARDS - acute respiratory distress syndrome; GI – gastrointestinal.

### Additional analyses

Models were fitted to estimate all treatment effects specified in the SAP (risk difference, RD; odds ratio, OR) for the primary and secondary outcomes. The results of these analyses are shown in Supplemental Table 10). The point estimates and confidence intervals are consistent with the primary results.

## Discussion

### Summary of main findings

The COMICS trial is the largest randomized trial of MiECC compared to CECC. MiECC reduced the frequency of SAEs prespecified to qualify for the primary outcome. This finding was of borderline significance due to stopping recruitment early but is consistent with the results of large-scale, published meta-analyses.^9,17^

MiECC improved a visual analogue quality-of-life measure. MiECC was safe with respect to other SAEs and adverse events that were reported. It did not reduce mortality, any SAE not included in the primary outcome, time to ICU or hospital discharge or transfusion of any red cells or any other blood product. However, all treatment effect estimates for these outcomes, except for hospital stay, favoured MiECC and the magnitude of the reductions in mortality and risk of any SAE not included in the primary outcome were consistent with the reduction in risk observed for the primary outcome.

### Findings in the context of the literature

Meta-analyses have considered various outcomes, e.g. MI, stroke, etc. The primary outcome in this trial was a composite of SAEs including death. The magnitude of the treatment effect for the primary outcome was clinically important and consistent with effects observed previously. This trial was not large enough to estimate treatment effects for component primary outcome events but, except for stage 3 AKI, there were fewer of each event in the MiECC than the CECC group (Supplemental Table 5). Transfusion of blood products is another outcome investigated in meta-analyses. Unlike the finding from another relatively large RCT,^18^ the difference overall was not statistically significant. However, the need to analyse *any* RBC transfusion, rather than *volumes* transfused, may have obscured significantly more or larger volume transfusions in the CECC group. Very similar percentages of patients in both groups had other blood products (platelets, FFP or cryoprecipitate) transfused.

Quality-of-life was assessed in a previous trial of MiECC versus CECC,^19^ which reported statistically significant differences in the Physical and Mental Component Scores of the Short Form 36 item instrument. In this trial, there was also a highly statistically significant benefit of MiECC compared to CECC in the visual analogue score of the EQ-5D-5L. The clinical importance of the observed difference to patients (2.6 points on a scale of 0 to 100) is uncertain. There was no difference between groups in the proportion of patients with an EQ-5D-5L index score of 1.

The trial was pragmatic, unlike many previous trials. A key feature was that any type II, III or IV MiECC could be used, rather than a specific product, reflecting a range of perfusion technology in clinical practice. COMICS allowed centres to use several components of optimized conventional circuits and contemporary monitoring and perfusion techniques (goal-directed perfusion, point-of-care coagulation and individual heparin management).^19^ Coated tubing was used for more than half, retrograde autologous priming for about a third and a centrifugal pump for about a quarter, of operations in patients in the CECC group. These and other aspects of CECC represent convergence towards MiECC, which would have tended to minimise differences between groups.

The primary outcome was less frequent than expected, based on an earlier trial.^15^ One reason may be that CECC in this trial used modern features not adopted 5-10 years ago (see above), such that the MiECC and CECC technologies had converged, despite the protocol requirement that CECC should not use a soft-shell reservoir or assisted drainage. Another reason may be that COMICS participants had a lower average EuroSCORE II than participants in the previous trial.

### Strengths and limitations

This trial was designed to address limitations affecting other trials of MiECC. It studied MiECC systems that had specific features, rather than specific commercial system(s). It was designed to be pragmatic and multicentre, with a clinically important primary outcome objectively documented or adjudicated. Randomized patients did not know their allocation. Data were analysed and reported according to a pre-specified statistical analysis plan.

The main limitation is the smaller than planned sample size due to early stopping of recruitment. Recruitment at the time had decreased due to a new wave of COVID-19 infections in the fall of 2021 and, even at its peak (about 50 randomized patients per month), a further 4 years would have been required to recruit the additional 2400-2500 randomized patients required to achieve the target. The TSC considered this not to be feasible given uncertainty about future recruitment.

Other limitations include small percentages of missing primary outcome data (3.0%, 1039/1071) and patients who contributed to the primary analysis but who did not receive their allocated intervention (1.9%, 20/1039) or for whom, additionally, the intervention did not adhere to the protocol (1.2%, 12/1019). At the outset, we did not consider it feasible to mandate all sites to use a single transfusion threshold; stratifying the analyses by site adjusted for sites using different thresholds but could not control for variable adherence to the nominated threshold between groups. A final limitation is potential bias due to the patients who were randomized but who could not be included in the primary analysis (14 in the CECC group and 18 in the MiECC group); among the 1071 randomized patients, 9 CECC patients and 29 MiECC patients did not receive their allocated intervention, whereas these numbers were 3 and 17 in the primary analysis.

Overall, a small number of patients did not have their allocated intervention. Most were patients in the MiECC group who in fact had CECC. ITT analysis ensured that no bias was introduced, i.e. even if such instances were the result of concern with MiECC, or reluctance to use MiECC for a particular patient, they were analysed ‘as if’ they had had MiECC.

### Conclusion

MiECC reduced the frequency of SAEs compared to CECC. This finding was of borderline significance due to early termination of the trial. MiECC improved quality-of-life as measured by the EQ-5D-5L visual analogue score. MiECC was safe with respect to other SAEs and adverse events that were reported. Continuing convergence of CECC and MiECC technologies may make future, adequately powered, trials of MiECC challenging because any difference in outcome between technologies is expected to decrease.

## Supplemental Material

Supplemental Material - Conventional versus minimally invasive extra-corporeal circulation in patients undergoing cardiac surgery: A randomized controlled trial (COMICS) Supplemental Material for Conventional versus minimally invasive extra-corporeal circulation in patients undergoing cardiac surgery: A randomized controlled trial (COMICS) by Gianni D Angelini, Barnaby C Reeves, Lucy A Culliford, Rachel Maishman, Chris A Rogers, Kyriakos Anastasiadis, Polychronis Antonitsis, Helena Argiriadou, Thierry Carrel, Dorothée Keller, Andreas Liebold, Fatma Ashkaniani, Aschraf El-Essawi, Ingo Breitenbach, Clinton Lloyd, Mark Bennett, Alex Cale, Serdar Gunaydin, Eren Gunertem, Farouk Oueida, Ibrahim M Yassin, Cyril Serrick, John M Murkin, Vivek Rao, Marco Moscarelli, Ignazzo Condello, Prakash Punjabi, Cha Rajakaruna, Apostolos Deliopoulos, Daniel Bone, William Lansdown, Narain Moorjani, Sarah Dennis in Perfusion
