# Supplementary material for: Conventional versus minimally invasive extra-corporeal circulation in patients undergoing cardiac surgery: A randomized controlled trial (COMICS)
Source: Perfusion. 2024 Jun 4;40(3):730–41. doi: 10.1177/02676591241258054 (PMC11951381; doi:10.1177/02676591241258054)
Supplement: Supplemental Material - Conventional versus minimally invasive extra-corporeal circulation in patients undergoing cardiac surgery: A randomized controlled trial (COMICS) [file sj-pdf-1-prf-10.1177_02676591241258054.pdf]

Table ST1 Protocol deviations

	Randomised to CECC (n=536)		Randomised to MiECC (n=535)		Overall (n=1071)	
	n	%	N	%	n	%
Did not receive the allocated treatment	9/529	1.7%	30/533	5.6%	39/1062	3.7%
Did not receive either allocation	*7/9*	*77.8%*	*13/30*	*43.3%*	*20/39*	*51.3%*
Received the alternative allocation	*2/9*	*22.2%*	*17/30*	*56.7%*	*19/39*	*48.7%*
Emergency or salvage operation	9/525	1.7%	6/526	1.1%	15/1051	1.4%
Underwent 3 procedures	1/525	0.2%	3/526	0.6%	4/1051	0.4%
Roller pump with MiECC			9/519	1.7%		
Soft shell reservoir with CECC	1/522	0.2%				
Assisted drainage with CECC	2/521	0.4%				

Reasons given for patient receiving the alternative allocation (n=1 unless otherwise stated):

CECC group: site entered incorrect allocation on A2 (site believed intervention given correctly) (n=2)

MiECC group: surgeon decision (no further details) (n=3);

"originally CABG alone, intraoperative TOE discovered at least moderate aortic stenosis. Surgeon decided to perform combined CABG and AVR utilizing standard CEC.";

site entered incorrect allocation on A2 (site believed intervention given correctly) (n=2);

"Decision by the surgeon, complex Operation LAA closure and PFO closure";

"Patient surgery rescheduled on a day where trained staff were not available.";

"Surgeon declined due to patient size";

"Time of surgery changed and no Perfusionist available for the new time slot.";

"Component in MiECC not available, unable to use, patient was operated via CECC";

"Original surgeon unable to operate, moved to non study surgeon who does not operate using MiECC. Operation done with CECC and non-study surgeon.";

"Operation required CECC (CABG, n=4, TVR, PFO closure, removal Lt Atrial Appendage).";

"No available MiECC trained personnel for CPB.";

"CABG+AVR Switch to CECC";

"Intraoperative Switch to CECC because of technical problem";

"switch to CECC because of a ventricular aneurysm"

Table ST2 Withdrawals

	Randomised to CECC (n=536)		Randomised to MiECC (n=535)		Overall (n=1071)	
	n	%	n	%	n	%
**Any withdrawal**	14/536	2.6%	12/535	2.2%	26/1071	2.4%
Decision taken by						
Patient	1/14	7.1%	2/12	16.7%	3/26	11.5%
Clinician	13/14	92.9%	10/12	83.3%	23/26	88.5%
Reason for withdrawal						
Treatment allocation	9/14	64.3%	9/12	75.0%	18/26	69.2%
Patient found to be ineligible after randomisation	1/14	7.1%	3/12	25.0%	4/26	15.4%
No reason given	1/14	7.1%	1/12	8.3%	2/26	7.7%
Surgery no longer required	2/14	14.3%	0/12	0.0%	2/26	7.7%
Other	11/14	78.6%	9/12	75.0%	20/26	76.9%
Timing of withdrawal						
After randomisation but before intervention	10/14	71.4%	12/12	100.0%	22/26	84.6%
After intervention	4/14	28.6%	0/12	0.0%	4/26	15.4%

There were an additional 11 pre-randomisation withdrawals. All patients were withdrawn due to clinician decision. Reasons given were (n=1 unless otherwise stated):

Operating surgeon changed to a non-participating surgeon or one able to use only CECC (n=5);

Clinician opted for conventional circuit;

Patient required additional procedure outside of CABG or AVR, no longer eligible;

Would not be best to use MiECC due to weight;

TCI date moved, missed, patient in theatre before randomised;

Was not randomised, before surgery, due to missing case report forms;

Study on hold due to COVID.

Table ST3 **Patient characteristics, medical history, pre-operative medications and pre-operative details**
(Note that information from Table1 is included)

	Randomised to CECC (n=536)		Randomised to MiECC (n=535)		Overall (n=1071)	
	n	%	n	%	n	%
**Demography**						
Male gender	440/525	83.8%	433/526	82.3%	873/1051	83.1%
Age (years, median, IQR)[1]	67.0	(58.0, 72.0)	66.0	(60.0, 73.0)	66.0	(59.0, 73.0)
BMI (median, IQR)[1]	28.10	(25.50, 31.00)	28.00	(25.30, 30.90)	28.10	(25.40, 31.00)
**Medical history**						
Renal impairment						
Normal (CC>85ml/min)	320/525	61.0%	324/526	61.6%	644/1051	61.3%
Moderate (CC>50 & <85)	178/525	33.9%	176/526	33.5%	354/1051	33.7%
Severe (CC<50)	24/525	4.6%	21/526	4.0%	45/1051	4.3%
Dialysis (regardless of CC)	3/525	0.6%	5/526	1.0%	8/1051	0.8%
Extracardiac arteriopathy	97/525	18.5%	96/526	18.3%	193/1051	18.4%
Active endocarditis	3/525	0.6%	2/526	0.4%	5/1051	0.5%
Poor mobility	23/525	4.4%	18/526	3.4%	41/1051	3.9%
Critical preoperative state	5/525	1.0%	7/526	1.3%	12/1051	1.1%
Previous cardiac surgery	5/525	1.0%	6/526	1.1%	11/1051	1.0%
Diabetes on insulin	98/525	18.7%	82/526	15.6%	180/1051	17.1%
Chronic pulmonary lung disease	56/525	10.7%	71/526	13.5%	127/1051	12.1%
NYHA						
I	136/525	25.9%	145/526	27.6%	281/1051	26.7%
II	272/525	51.8%	267/526	50.8%	539/1051	51.3%
III	107/525	20.4%	104/526	19.8%	211/1051	20.1%
IV	10/525	1.9%	10/526	1.9%	20/1051	1.9%
CCS						
0	143/499	28.7%	141/503	28.0%	284/1002	28.3%
I	95/499	19.0%	95/503	18.9%	190/1002	19.0%
II	159/499	31.9%	157/503	31.2%	316/1002	31.5%

	Randomised to CECC (n=536)		Randomised to MiECC (n=535)		Overall (n=1071)	
	n	%	n	%	n	%
III	58/499	11.6%	62/503	12.3%	120/1002	12.0%
IV	44/499	8.8%	48/503	9.5%	92/1002	9.2%
LV function						
Good (LVEF >50%)	366/525	69.7%	375/526	71.3%	741/1051	70.5%
Moderate (LVEF 31% - 50%)	145/525	27.6%	138/526	26.2%	283/1051	26.9%
Poor (LVEF 21% - 30%)	12/525	2.3%	12/526	2.3%	24/1051	2.3%
Very poor (LVEF <20%)	2/525	0.4%	1/526	0.2%	3/1051	0.3%
>50% disease in left main stem	113/522	21.6%	123/526	23.4%	236/1048	22.5%
Recent MI	101/525	19.2%	98/526	18.6%	199/1051	18.9%
Pulmonary hypertension						
Moderate (PA systolic 31-55mmHg)	26/523	5.0%	30/526	5.7%	56/1049	5.3%
Severe (PA systolic >55mmHg)	4/523	0.8%	3/526	0.6%	7/1049	0.7%
None	493/523	94.3%	493/526	93.7%	986/1049	94.0%
EuroSCORE II (median, IQR)	1.25	(0.81, 2.08)	1.22	(0.84, 2.04)	1.24	(0.83, 2.05)
Smoking history						
Non-smoker	211/523	40.3%	222/526	42.2%	433/1049	41.3%
Ex-smoker >1 month	192/523	36.7%	169/526	32.1%	361/1049	34.4%
Current smoker	120/523	22.9%	135/526	25.7%	255/1049	24.3%
Family history (cardiac)	201/524	38.4%	215/525	41.0%	416/1049	39.7%
Diabetes						
None	301/524	57.4%	338/526	64.3%	639/1050	60.9%
Diet	15/524	2.9%	12/526	2.3%	27/1050	2.6%
Oral	109/524	20.8%	91/526	17.3%	200/1050	19.0%
Insulin	99/524	18.9%	85/526	16.2%	184/1050	17.5%
Hypercholesterolaemia	407/524	77.7%	420/526	79.8%	827/1050	78.8%
Hypertension requiring treatment	424/524	80.9%	436/526	82.9%	860/1050	81.9%
CVA/TIAs	33/524	6.3%	36/526	6.8%	69/1050	6.6%
Neurological dysfunction	10/524	1.9%	9/526	1.7%	19/1050	1.8%
Severe neurological disease	4/524	0.8%	4/526	0.8%	8/1050	0.8%

	Randomised to CECC (n=536)		Randomised to MiECC (n=535)		Overall (n=1071)	
	**n**	**%**	**n**	**%**	**n**	**%**
Peripheral vascular disease	71/524	13.5%	73/526	13.9%	144/1050	13.7%
Post-MI Ventricular Septal Defect (VSD)	1/524	0.2%	5/526	1.0%	6/1050	0.6%
Heart rhythm						
Sinus	494/524	94.3%	487/526	92.6%	981/1050	93.4%
AF	25/524	4.8%	32/526	6.1%	57/1050	5.4%
Block	5/524	1.0%	7/526	1.3%	12/1050	1.1%
Unstable angina	57/485	11.8%	61/492	12.4%	118/977	12.1%
Congestive heart failure	12/524	2.3%	10/526	1.9%	22/1050	2.1%
**Preoperative medications**						
Heparin (fractionated/unfractionated)	134/524	25.6%	150/526	28.5%	284/1050	27.0%
Prophylactic	118/134	88.1%	128/150	85.3%	246/284	86.6%
Treatment	16/134	11.9%	22/150	14.7%	38/284	13.4%
Potassium channel activators	15/524	2.9%	10/526	1.9%	25/1050	2.4%
Anti-arrhythmic	22/524	4.2%	29/526	5.5%	51/1050	4.9%
Antiplatelet (P2Y12 inhibitors)	129/524	24.6%	131/526	24.9%	260/1050	24.8%
Statins	420/524	80.2%	424/526	80.6%	844/1050	80.4%
Warfarin	9/524	1.7%	4/526	0.8%	13/1050	1.2%
Other lipid lowering agent (exc. statins)	52/524	9.9%	64/526	12.2%	116/1050	11.0%
NOAC (new oral anticoagulants)	32/524	6.1%	31/526	5.9%	63/1050	6.0%
ACE inhibitors	244/524	46.6%	226/526	43.0%	470/1050	44.8%
Beta blockers	367/524	70.0%	357/526	67.9%	724/1050	69.0%
Angiotensin II blockers	96/524	18.3%	122/526	23.2%	218/1050	20.8%
Calcium channel blockers	152/524	29.0%	143/526	27.2%	295/1050	28.1%
Diuretics	142/524	27.1%	126/526	24.0%	268/1050	25.5%
Nitrates	112/524	21.4%	111/526	21.1%	223/1050	21.2%
Aldosterone antagonists	33/524	6.3%	29/526	5.5%	62/1050	5.9%
Alpha adrenoreceptor blocker	23/524	4.4%	22/526	4.2%	45/1050	4.3%
Aspirin Not P2Y12	268/368	72.8%	264/360	73.3%	532/728	73.1%

	Randomised to CECC (n=536)		Randomised to MiECC (n=535)		Overall (n=1071)	
	n	%	n	%	n	%
**Angiogram/Echo results**						
Coronary disease, number of vessels						
None	45/525	8.6%	40/526	7.6%	85/1051	8.1%
Single	19/525	3.6%	21/526	4.0%	40/1051	3.8%
Double	84/525	16.0%	76/526	14.4%	160/1051	15.2%
Triple	366/525	69.7%	379/526	72.1%	745/1051	70.9%
Not investigated	11/525	2.1%	10/526	1.9%	21/1051	2.0%
Aortic stenosis						
None	426/520	81.9%	418/524	79.8%	844/1044	80.8%
Mild	20/520	3.8%	28/524	5.3%	48/1044	4.6%
Moderate	7/520	1.3%	7/524	1.3%	14/1044	1.3%
Severe	67/520	12.9%	71/524	13.5%	138/1044	13.2%
Aortic regurgitation						
None	428/519	82.5%	424/524	80.9%	852/1043	81.7%
Mild	70/519	13.5%	70/524	13.4%	140/1043	13.4%
Moderate	14/519	2.7%	18/524	3.4%	32/1043	3.1%
Severe	7/519	1.3%	12/524	2.3%	19/1043	1.8%
Mitral regurgitation						
None	361/519	69.6%	335/524	63.9%	696/1043	66.7%
Mild	144/519	27.7%	179/524	34.2%	323/1043	31.0%
Moderate	13/519	2.5%	9/524	1.7%	22/1043	2.1%
Severe	1/519	0.2%	1/524	0.2%	2/1043	0.2%
Haemoglobin (mean, SD)[1]	13.88	1.72	13.94	1.64	13.91	1.68
Platelets (mean, SD)[1]	239.88	73.43	235.81	70.17	237.84	71.81

[1] missing for 20 patients (11 randomised to CECC and 9 randomised to MiECC)

Table ST4 Details of operation, biochemistry and clinical observations up to 24 hours after surgery

	Randomised to CECC (n=536)		Randomised to MiECC (n=535)		Overall (n=1071)	
	n	%	n	%	n	%
**Operative details**						
Number of distal coronary anastomoses (median, IQR) (CABG only)[1]	3	(3.0, 3.0)	3	(3.0, 4.0)	3	(3.0, 4.0)
Prosthetic valve						
Mechanical	24/82	29.3%	17/83	20.5%	41/165	24.8%
Tissue	57/82	69.5%	66/83	79.5%	123/165	74.5%
N/A	1/82	1.2%	0/83	0.0%	1/165	0.6%
**Myocardial protection**						
Cardioplegia solution						
Blood	421/523	80.5%	422/519	81.3%	843/1042	80.9%
Crystalloid	36/523	6.9%	35/519	6.7%	71/1042	6.8%
Other	66/523	12.6%	62/519	11.9%	128/1042	12.3%
Temperature						
Warm	331/522	63.4%	341/519	65.7%	672/1041	64.6%
Cooled	191/522	36.6%	178/519	34.3%	369/1041	35.4%
Infusion mode						
Antegrade	462/522	88.5%	461/519	88.8%	923/1041	88.7%
Retrograde and antegrade	60/522	11.5%	58/519	11.2%	118/1041	11.3%
Timing						
Intermittent	516/522	98.9%	506/519	97.5%	1022/1041	98.2%
Continuous	6/522	1.1%	13/519	2.5%	19/1041	1.8%
**Intraoperative details**						
Lowest haematocrit (%) (mean, SD)[2]	26.21	6.80	28.55	6.43	27.38	6.72
Lowest core temperature (°C) (mean, SD)[3]	34.90	(33.8, 35.8)	35.0	(34.0, 35.8)	35.0	(33.9, 35.8)
Intraoperative inotropes	184/523	35.2%	185/520	35.6%	369/1043	35.4%
Intraoperative noradrenalin	328/523	62.7%	321/520	61.7%	649/1043	62.2%

	Randomised to CECC (n=536)		Randomised to MiECC (n=535)		Overall (n=1071)	
	n	%	n	%	n	%
Intraoperative vasodilator	88/523	16.8%	88/520	16.9%	176/1043	16.9%
Intraoperative IABP	15/524	2.9%	17/520	3.3%	32/1044	3.1%
**Cardiopulmonary bypass details**						
**Type of circuit**						
Circuit						
CECC	520/522	99.6%	17/520	3.3%	537/1042	51.5%
MiECC	2/522	0.4%	503/520	96.7%	505/1042	48.5%
MiECC circuit type						
II	1/2	50.0%	175/501	34.9%	176/503	35.0%
III	0/2	0.0%	135/501	26.9%	135/503	26.8%
IV	1/2	50.0%	191/501	38.1%	192/503	38.2%
If type IV, was conversion to open circuit required?	0/1	0.0%	2/190	1.1%	2/191	1.0%
Total bypass time (minutes) (mean, SD)[4]	87.3	30.4	89.3	30.1	88.3	30.3
Cumulative cross clamp time (minutes) (mean, SD)[4]	56.4	21.4	57.0	21.0	56.7	21.2
Noradrenaline use during ECC	249/521	47.8%	242/518	46.7%	491/1039	47.3%
Neosynephrine use during ECC	107/520	20.6%	63/518	12.2%	170/1038	16.4%
**CECC/MiECC components**						
Coating	455/520	87.5%	499/517	96.5%	954/1037	92.0%
Reservoir						
Hard shell	521/522	99.8%	170/515	33.0%	691/1037	66.6%
Soft shell	1/522	0.2%	326/515	63.3%	327/1037	31.5%
None	0/522	0.0%	19/515	3.7%	19/1037	1.8%
Pump						
Roller	395/522	75.7%	9/519	1.7%	404/1041	38.8%
Centrifugal	127/522	24.3%	510/519	98.3%	637/1041	61.2%
Coated tubing	296/520	56.9%	491/518	94.8%	787/1038	75.8%
Priming volume (ml) (median, IQR)[5]	1250	(1200, 1500)	750	(600, 800)	1035.0	(750, 1250)
Retrograde autologous priming	169/522	32.4%	287/519	55.3%	456/1041	43.8%

	Randomised to CECC (n=536)		Randomised to MiECC (n=535)		Overall (n=1071)	
	n	%	n	%	n	%
Venting						
None	29/506	5.7%	25/498	5.0%	54/1004	5.4%
Right superior pulmonary vein	86/506	17.0%	96/498	19.3%	182/1004	18.1%
Pulmonary artery	50/506	9.9%	79/498	15.9%	129/1004	12.8%
Left ventricle	48/506	9.5%	31/498	6.2%	79/1004	7.9%
Aortic root	293/506	57.9%	267/498	53.6%	560/1004	55.8%
Cardiotomy suction	462/522	88.5%	185/519	35.6%	647/1041	62.2%
Cell saver set up	134/521	25.7%	361/519	69.6%	495/1040	47.6%
If yes, processed volume infused (median, IQR)[6]	236	(0.0, 400)	259.0	(149.0, 500)	250.0	(124, 487)
Unprocessed volume infused[7]	0.0	(0.0, 0.0)	0.0	(0.0, 0.0)	0.0	(0.0, 0.0)
Total crystalloid volume given (median, IQR)[8]	1500.0	(999, 3000	1400.0	(600, 2600)	1500.0	(750, 2800)
Total colloid volume given (median, IQR)[9]	0.0	(0, 250)	0.0	(0.0, 250)	0.0	(0, 250)
Venous drainage						
Passive	519/521	99.6%	73/515	14.2%	592/1036	57.1%
Assisted	2/521	0.4%	442/515	85.8%	444/1036	42.9%
Heparin dosage						
300 IU/kg	477/478	99.8%	423/475	89.1%	900/953	94.4%
150 IU/kg	1/478	0.2%	52/475	10.9%	53/953	5.6%
Target ACT (minutes) (median, IQR)[5]	480	(400, 480)	400	(400, 480)	467.0	(400, 480)
**Post-operative details**						
HCT on return from theatre (%) (mean, SD)[10]	31.30	5.78	31.77	5.72	31.53	5.75
Lactate on return from theatre (mmol/l) (median, IQR)[11]	1.50	(1.00, 2.50)	1.40	(1.00, 2.30)	1.40	(1.00, 2.40)
Temperature on return from theatre (°C) (median, IQR)[12]	36.00	(35.5, 36.5)	36.00	(35.5, 36.5)	36.00	(35.5, 36.5)
Lowest Hb during 1st 24 hours (g/dl) (mean, SD)[13]	9.35	1.78	9.56	2.12	9.45	1.96
Lowest HCT during 1st 24 hours (%) (mean, SD)[14]	28.27	5.07	28.58	5.22	28.43	5.14
Lowest MABP during 1st 24 hours (mmHg) (mean, SD) [14]	65.9	10.5	66.3	11.5	66.1	11.0
Highest lactate during 1st 24 hours (mmol/l) (mean, SD)[13]	2.40	(1.50, 3.90)	2.20	(1.40, 3.80)	2.30	(1.40, 3.90)

[1] missing for 16 patients (5 randomised to CECC and 11 to MiECC)

[2] missing for 30 patients (13 randomised to CECC and 17 to MiECC)

[3] missing for 60 patients (29 randomised to CECC and 31 to MiECC)

[4] missing for 31 patients (14 randomised to CECC and 17 to MiECC)

[5] missing for 30 patients (14 randomised to CECC and 16 to MiECC)

[6] missing for 7 patients (2 randomised to CECC and 6 to MiECC)

[7] missing for 23 patients (8 randomised to CECC and 15 to MiECC)

[8] missing for 213 patients (102 randomised to CECC and 111 to MiECC)

[9] missing for 214 patients (102 randomised to CECC and 112 to MiECC)

[10] missing for 109 patients (55 randomised to CECC and 54 to MiECC)

[11] missing for 27 patients (14 randomised to CECC and 13 to MiECC)

[12] missing for 34 patients (18 randomised to CECC and 16 to MiECC)

[13] missing for 28 patients (15 randomised to CECC and 13 to MiECC)

[14] missing for 30 patients (16 randomised to CECC and 14 to MiECC)

Table ST5 Frequencies of primary and secondary outcomes and estimated treatment effects for CECC vs MiECC groups.

	**Randomised to CECC (n=536)**		**Randomised to MiECC (n=535)**		**Estimate[1] (95% CI)**	**p-value**
	**n**	**%**	**N**	**%**		
					RR	
**Experienced a primary outcome event[2]**	69/522	13.2%	50/517	9.7%	0.732 (0.556, 0.962)	0.025
Primary outcome events:						
**Death**	10/524	1.9%	8/522	1.5%	0.796 (0.364, 1.740)	0.568
Myocardial infarction	4/522	0.8%	1/522	0.2%		
Stroke	6/522	1.1%	0/522	0.0%		
Gut infarction	1/522	0.2%	0/522	0.0%		
Stage 3 AKI including haemofiltration[3]	10/522	1.9%	13/522	2.5%		
Reintubation[4]	26/522	5.0%	13/522	2.5%		
Tracheostomy	6/522	1.1%	3/522	0.6%		
Mechanical ventilation for >48 hours[5]	14/519	2.7%	13/518	2.5%		
Reoperation	21/522	4.0%	17/522	3.3%		
Percutaneous intervention	2/522	0.4%	1/522	0.2%		
Sternal wound infection with dehiscence[6]	11/522	2.1%	3/522	0.6%		
Septicaemia confirmed by microbiology	10/522	1.9%	3/522	0.6%		
**Any other SAE (not included in primary outcome)**	68/509	13.4%	53/504	10.5%	0.791 (0.530, 1.179)	0.250
**Blood products transfused during hospital stay**						
Any red cell transfusion[7]	201/521	38.6%	168/518	32.4%	0.839 (0.695, 1.012)	0.067
Any other blood product transfusion[7]	55/521	10.6%	58/518	11.2%	1.067 (0.806, 1.414)	0.650
					HR	
**Time to ICU discharge (hours) (median, IQR)[8]**	23.95	(20.7, 69.0)	24.0	(21.0,67.0)	0.98 (0.87, 1.11)	0.781
**Time to hospital discharge (days) (median, IQR)[9]**	7.0	(6.0, 8.0)	7.0	(6.0, 8.0)	1.05 (0.93, 1.19)	0.420
**EQ5D 5L index score (median, IQR)**					RR	
Baseline[10]	0.77	(0.65, 0.91)	0.80	(0.66, 1.00)		
30 days[10]	0.78	(0.68, 0.84)	0.81	(0.73, 0.91)	0.96 (0.90 to 1.02)[11,12]	0.151
90 days[10]	0.88	(0.80, 1.00)	0.91	(0.84, 1.00)		
**EQ5D 5L VAS score (mean, SD)**					MD	
Baseline[10]	68.7	(17.3)	69.7	(17.5)		
30 days[10]	73.3	(14.9)	76.6	(13.0)	2.56 (1.22 to 3.89)[12]	<0.001
90 days[10]	81.9	(12.7)	84.1	(12.1)		

Prespecified outcomes are in bold font.

[1] Treatment effects: RR=Risk ratio, HR=Hazard ratio, MD=mean difference

[2] Participants could experience multiple events. ECGs were not available for suspected MIs and suspected MIs were confirmed (serum troponin >500 ng/L) for only 5 patients. In addition, 1 participant in the CECC group and 5 participants in the MiECC group had a suspected MI with serum troponin <500. Including all patients with a suspected MI as having the primary outcome did not change the treatment effect (RR=0.77,95% CI 0.59, 1.00, p=0.048).

[3] 9 participants (4 randomised to CECC, 5 randomised to MiECC) with haemofiltration only were classified as stage 3 AKI

[4] 2 events of reintubation in one participant in the MiECC treatment group

[5] Defined as having mechanical ventilation continuing after post-operative day 2. 1 participant with prolonged ventilation SAE in the MiECC group received the alternative treatment.

[6] 2 events of wound dehiscence in one participant in the CECC treatment group

[7] The statistical analysis plan erroneously defined the transfusion outcomes as post-op transfusions only. The initial analysis shared with investigators used this definition. After seeing the transfusion percentages for intra- and post-op RBC transfusion and checking the definition in the protocol (which refers to any RBC transfusion) the SAP was corrected, the analysis code revised and the model re-run.

[8] missing for 34 patients (17 randomised to CECC and 17 to MiECC)

[9] missing for 25 patients (12 randomised to CECC and 13 to MiECC)

[10] missing at baseline for 27 patients (16 randomised to CECC and 11 to MiECC); at 30 days for 94 patients (53 randomised to CECC and 41 to MiECC); at 90 days for 106 patients (56 randomised to CECC and 50 to MiECC)

[11] RR (risk ratio) is the risk of less than perfect health, i.e. index score <1

[12] Interaction of treatment by time was tested but was not significant and was excluded from the model

Table ST6 Blood products transfused during hospital stay

	Randomised to CECC (n=536)		Randomised to MiECC (n=535)		Overall (n=1071)	
	n	%	n	%	n	%
**Intraoperative transfusion of blood product**						
RBC used	113/521	21.7%	62/519	11.9%	175/1040	16.8%
1 unit transfused	52/521	10.0%	35/519	6.7%	87/1040	8.4%
2 units transfused	51/521	9.8%	24/519	4.6%	75/1040	7.2%
3+ units transfused	10/521	1.9%	3/519	0.6%	13/1040	1.3%
FFP used	22/521	4.2%	12/519	2.3%	34/1040	3.3%
1 unit transfused	14/521	2.7%	6/519	1.2%	20/1040	1.9%
2 units transfused	6/521	1.2%	4/519	0.8%	10/1040	1.0%
3+ units transfused	2/521	0.4%	2/519	0.4%	4/1040	0.4%
Platelets used	19/521	3.6%	12/519	2.3%	31/1040	3.0%
1 unit transfused	8/521	1.5%	6/519	1.2%	14/1040	1.3%
2 units transfused	3/521	0.6%	5/519	1.0%	8/1040	0.8%
3+ units transfused	8/521	1.5%	1/519	0.2%	9/1040	0.9%
Cryoprecipitate used	2/521	0.4%	0/519	0.0%	2/1040	0.2%
3+ units transfused	2/521	0.4%	0/519	0.0%	2/1040	0.2%
**Post-operative transfusion of blood product**						
Red blood cells	156/522	29.9%	139/520	26.7%	295/1042	28.3%
1 unit transfused	75/522	14.4%	62/520	11.9%	137/1042	13.1%
2 units transfused	52/522	10.0%	47/520	9.0%	99/1042	9.5%
3+ units transfused	29/522	5.6%	30/520	5.8%	59/1042	5.7%
FFP	18/522	3.4%	24/520	4.6%	42/1042	4.0%
Platelets	13/522	2.5%	21/520	4.0%	34/1042	3.3%
Cryoprecipitate	1/522	0.2%	2/520	0.4%	3/1042	0.3%
**Transfusion of any blood product during hospital stay**						
RBC	201/521	38.6%	168/518	32.4%	369/1039	35.5%
1 unit transfused	68/521	13.1%	60/518	11.6%	128/1039	12.3%
2 units transfused	66/521	12.7%	65/518	12.5%	131/1039	12.6%
3+ units transfused	67/521	12.9%	43/518	8.3%	110/1039	10.6%
FFP	36/520	6.9%	35/517	6.8%	71/1037	6.8%
Platelets	28/520	5.4%	29/518	5.6%	57/1038	5.5%
Cryoprecipitate	3/519	0.6%	2/517	0.4%	5/1036	0.5%
Any other (FFP, platelets or cryoprecipitate)	55/521	10.6%	58/518	11.2%	113/1039	10.9%

Table ST7 Longitudinal EQ-5D-5L quality of life outcomes and treatment effects (index and visual analogue scores)

EQ5D 5L index score	Randomised to CECC (n=536)		Randomised to MiECC (n=535)		Overall (n=1071)	
	**Median**	**IQR**	**Median**	**IQR**	**Median**	**IQR**
Baseline[3]	0.77	(0.65, 0.91)	0.80	(0.66, 1.00)	0.78	(0.66, 0.91)
30 days[4]	0.78	(0.68, 0.84)	0.81	(0.73, 0.91)	0.80	(0.71, 0.88)
90 days[5]	0.88	(0.80, 1.00)	0.91	(0.84, 1.00)	0.88	(0.80, 1.00)
Treatment*time interaction (RR, 95% CI)					RR[1]	0.5166
Overall treatment effect (RR, 95% CI)					0.96	(0.90 to 1.02)

EQ5D 5L VAS score	Randomised to CECC (n=536)		Randomised to MiECC (n=535)		Overall (n=1071)	
	**Mean**	**SD**	**Mean**	**SD**	**Mean**	**SD**
Baseline[3]	68.7	(17.3)	69.7	(17.5)	69.2	(17.4)
30 days[4]	73.3	(14.9)	76.6	(13.0)	75.0	(14.1)
90 days[5]	81.9	(12.7)	84.1	(12.1)	83.0	(12.4)
Treatment*time interaction (MD, 95% CI)					MD[2]	0.129
Overall treatment effect (MD, 95% CI)					2.56	(1.22 to 3.89)

1. RR (risk ratio) for risk of less than perfect health, i.e. index score <1; RR<1 means a lower risk of having less than perfect health in the MiECC group than the CECC group (p-value for the comparison = 0.151).
2. MD (mean difference); the positive MD means a higher score in the MiECC group than the CECC group (p-value for the comparison < 0.001).
3. missing for 27 patients (16 randomised to CECC and 11 to MiECC)
4. missing for 94 patients (53 randomised to CECC and 41 to MiECC)
5. missing for 106 patients (56 randomised to CECC and 50 to MiECC)

Table ST8 Unexpected serious adverse events (SAEs) classified by Medical Dictionary of for Regulatory Activities (MedDRA) preferred term

Unexpected SAE by MedDRA preferred term	Randomised to CECC (n=536)		Randomised to MiECC (n=535)		Overall (n=1071)	
	n/N	%	n/N	%	n/N	%
**Any unexpected SAE**	28/515	5.4%	31/515	6.0%	59/1030	5.7%
**Total number of events**	43		43		86	
Admission reason unknown	1/515	0.2%	0/515	0.0%	1/1030	0.1%
Blood system disorders						
Anaemia	0/515	0.0%	1/515	0.2%	1/1030	0.1%
Blood loss anaemia	0/515	0.0%	1/515	0.2%	1/1030	0.1%
Cardiac disorders						
Atrial fibrillation	1/515	0.2%	1/515	0.2%	2/1030	0.2%
Atrioventricular block	0/515	0.0%	1/515	0.2%	1/1030	0.1%
Bradycardia	1/515	0.2%	0/515	0.0%	1/1030	0.1%
Cardiac failure	0/515	0.0%	1/515	0.2%	1/1030	0.1%
Cardiac tamponade	1/515	0.2%	0/515	0.0%	1/1030	0.1%
Low cardiac output syndrome	2/515	0.4%	1/515	0.2%	3/1030	0.3%
Pericardial effusion	1/515	0.2%	2/515	0.4%	3/1030	0.3%
Pericarditis	1/515	0.2%	0/515	0.0%	1/1030	0.1%
Right ventricular failure	1/515	0.2%	0/515	0.0%	1/1030	0.1%
Tachyarrhythmia	1/515	0.2%	0/515	0.0%	1/1030	0.1%
Ventricular tachycardia	1/515	0.2%	0/515	0.0%	1/1030	0.1%
Gastrointestinal disorders						
Abdominal pain	1/515	0.2%	0/515	0.0%	1/1030	0.1%
Constipation	0/515	0.0%	1/515	0.2%	1/1030	0.1%
Gastrointestinal haemorrhage	0/515	0.0%	1/515	0.2%	1/1030	0.1%
Gastrointestinal pain	0/515	0.0%	1/515	0.2%	1/1030	0.1%
Ileus paralytic	0/515	0.0%	1/515	0.2%	1/1030	0.1%
Upper gastrointestinal haemorrhage	1/515	0.2%	0/515	0.0%	1/1030	0.1%
Vomiting	1/515	0.2%	0/515	0.0%	1/1030	0.1%
General disorders and administration site conditions						
Chest pain	2/515	0.4%	3/515	0.6%	5/1030	0.5%
Fatigue	0/515	0.0%	1/515	0.2%	1/1030	0.1%
Illness	1/515	0.2%	0/515	0.0%	1/1030	0.1%
Multiple organ dysfunction syndrome	3/515	0.6%	1/515	0.2%	4/1030	0.4%
Pyrexia	1/515	0.2%	1/515	0.2%	2/1030	0.2%
Infections and infestations						
COVID-19	0/515	0.0%	1/515	0.2%	1/1030	0.1%
Pneumonia[1, 2]	1/515	0.2%	1/515	0.2%	2/1030	0.2%
Postoperative wound infection	1/515	0.2%	0/515	0.0%	1/1030	0.1%

Respiratory tract infection	1/515	0.2%	1/515	0.2%	2/1030	0.2%
Sepsis	2/515	0.4%	0/515	0.0%	2/1030	0.2%
Urinary tract infection	1/515	0.2%	0/515	0.0%	1/1030	0.1%
Wound infection	2/515	0.4%	1/515	0.2%	3/1030	0.3%
Injury, poisoning and procedural complications						
Fall	1/515	0.2%	0/515	0.0%	1/1030	0.1%
Postoperative thoracic procedure complication	1/515	0.2%	0/515	0.0%	1/1030	0.1%
Wound haemorrhage	0/515	0.0%	1/515	0.2%	1/1030	0.1%
Metabolism and nutrition disorders						
Dehydration	1/515	0.2%	0/515	0.0%	1/1030	0.1%
Arthralgia	0/515	0.0%	1/515	0.2%	1/1030	0.1%
Nervous system disorders						
Cerebral haemorrhage	1/515	0.2%	0/515	0.0%	1/1030	0.1%
Cerebral ischaemia	0/515	0.0%	1/515	0.2%	1/1030	0.1%
Dizziness	1/515	0.2%	1/515	0.2%	2/1030	0.2%
Headache	0/515	0.0%	1/515	0.2%	1/1030	0.1%
Seizure	0/515	0.0%	1/515	0.2%	1/1030	0.1%
Syncope	0/515	0.0%	1/515	0.2%	1/1030	0.1%
Renal and urinary disorders						
Acute kidney injury	0/515	0.0%	1/515	0.2%	1/1030	0.1%
Renal impairment	1/515	0.2%	0/515	0.0%	1/1030	0.1%
Urinary retention	0/515	0.0%	1/515	0.2%	1/1030	0.1%
Respiratory, thoracic and mediastinal disorders						
Dyspnoea	2/515	0.4%	3/515	0.6%	5/1030	0.5%
Pleural effusion[2]	2/515	0.4%	1/515	0.2%	3/1030	0.3%
Pneumothorax	0/515	0.0%	1/515	0.2%	1/1030	0.1%
Pulmonary embolism	0/515	0.0%	1/515	0.2%	1/1030	0.1%
Respiratory failure	0/515	0.0%	1/515	0.2%	1/1030	0.1%
Surgical and medical procedures						
Cardiac pacemaker insertion	1/515	0.2%	1/515	0.2%	2/1030	0.2%
High frequency ablation	1/515	0.2%	0/515	0.0%	1/1030	0.1%
Resuscitation	0/515	0.0%	1/515	0.2%	1/1030	0.1%
Wound closure	1/515	0.2%	0/515	0.0%	1/1030	0.1%
Vascular disorders						
Haemorrhage	0/515	0.0%	1/515	0.2%	1/1030	0.1%
Hypertension	1/515	0.2%	1/515	0.2%	2/1030	0.2%

[1] *2 events of pneumonia in one patient in the MiECC treatment group*

[2] *1 patient who experienced SAE in MiECC group received the alternative treatment.*

Table ST9 In-hospital post-operative adverse events (AEs)

	Randomised to CECC (n=536)		Randomised to MiECC (n=535)		Overall (n=1071)	
	n/N	%	n/N	%	n/N	%
Cardiac arrest	8/521	1.5%	6/517	1.2%	14/1038	1.3%
Attempt to resuscitate	7/8	87.5%	6/6	100.0%	13/14	92.9%
Resuscitation successful	5/7	71.4%	2/6	33.3%	7/13	53.8%
SVT/AF requiring treatment	90/521	17.3%	88/517	17.0%	178/1038	17.1%
VF/VT requiring intervention	5/521	1.0%	0/517	0.0%	5/1038	0.5%
New pacing	20/521	3.8%	24/517	4.6%	44/1038	4.2%
Single	10/20	50.0%	7/24	29.2%	17/44	38.6%
Double	10/20	50.0%	17/24	70.8%	27/44	61.4%
Temporary pacing became permanent	2/20	10.0%	5/24	20.8%	7/44	15.9%
Vasopressors used	177/520	34.0%	186/516	36.0%	363/1036	35.0%
Any inotropes used	262/520	50.4%	293/516	56.8%	555/1036	53.6%
IABP inserted	9/521	1.7%	14/517	2.7%	23/1038	2.2%
Pulmonary artery catheter inserted	9/521	1.7%	10/517	1.9%	19/1038	1.8%
Vasodilator used	33/521	6.3%	27/515	5.2%	60/1036	5.8%
Mask CPAP	32/521	6.1%	34/517	6.6%	66/1038	6.4%
ARDS	2/521	0.4%	2/517	0.4%	4/1038	0.4%
Pneumothorax or pleural effusion requiring drainage	16/521	3.1%	22/516	4.3%	38/1037	3.7%
Peptic ulcer/GI bleed/ perforation	0/520	0.0%	0/517	0.0%	0/1037	0.0%
Pancreatitis (amylase >1500iu)	0/520	0.0%	0/517	0.0%	0/1037	0.0%
Ischaemic bowel requiring treatment	1/520	0.2%	1/517	0.2%	2/1037	0.2%
Transient Ischaemic Attack (TIA)	3/520	0.6%	1/517	0.2%	4/1037	0.4%
Deep Vein Thrombosis (DVT)	0/520	0.0%	1/517	0.2%	1/1037	0.1%
Pulmonary embolus	0/520	0.0%	0/517	0.0%	0/1037	0.0%
Excess bleeding, not requiring re-operation	6/520	1.2%	11/517	2.1%	17/1037	1.6%
Pericardial effusion	4/520	0.8%	10/517	1.9%	14/1037	1.4%
Suspected sepsis	17/520	3.3%	15/517	2.9%	32/1037	3.1%
Temperature <36 or >38	13/17	76.5%	13/15	86.7%	26/32	81.3%
Unexplained increased heart rate above normal for patient	4/17	23.5%	4/15	26.7%	8/32	25.0%
CRP >5mg/L	10/17	58.8%	11/14	78.6%	21/31	67.7%
WBC >15.0 (10*9/L)	12/17	70.6%	7/14	50.0%	19/31	61.3%
Unexplained increased respiratory rate	6/17	35.3%	1/15	6.7%	7/32	21.9%
Respiratory infection	20/519	3.9%	19/515	3.7%	39/1034	3.8%
Superficial wound infection	13/519	2.5%	6/515	1.2%	19/1034	1.8%
Mediastinitis	1/519	0.2%	0/515	0.0%	1/1034	0.1%
UTI	1/519	0.2%	4/515	0.8%	5/1034	0.5%
Unspecified infection	7/519	1.3%	11/515	2.1%	18/1034	1.7%

Note: adverse events reported in this table that met the criteria of an SAE are also reported in Table 3

Table ST10 Alternative analyses of primary and secondary outcomes

Outcome	Treatment effect	Estimate (95% CI), p-value
Primary outcome	**Risk ratio**	0.732 (0.556, 0.962), p=0.025
	Odds ratio	0.702 (0.477, 1.034), p=0.073
	**Risk difference**	-0.035 (-0.074, 0.003), p=0.073
Death	**Risk ratio**	**0.796 (0.364, 1.740), p=0.568**
	Odds ratio	0.789 (0.306, 2.036), p=0.624
	Risk difference	-0.007 (-0.0249, 0.010), p=0.408
Any other SAE	**Risk ratio**	**0.791 (0.530, 1.179), p=0.250**
	Odds ratio	0.757 (0.512, 1.119), p=0.163
	Risk difference	-0.031 (-0.068, 0.005), p=0.096
Any other blood product transfusion	**Risk ratio**	0.839 (0.695, 1.012), p=0.067
	Odds ratio	0.739 (0.565, 0.967), p=0.027
	Risk difference	-0.0499 (-0.105, 0.005), p=0.077
Any RBC transfusion	**Risk ratio**	1.067 (0.806, 1.414), p=0.650
	Odds ratio	1.088 (0.7196, 1.644), p=0.690
	Risk difference	0.014 (-0.014, 0.043), p=0.326

The primary analyses reported in Table 2 are shown in bold. Models to estimate risk differences have been fitted with site as a random effect and the binomial identity matrix. For models to converge, the number of adaptive quadrature points had to be specified. Models using the binomial identity matrix with site as a cluster variable were also fitted to check these estimates (these models were fitted without the need for specifying quadrature points), with results consistent with the ones shown.

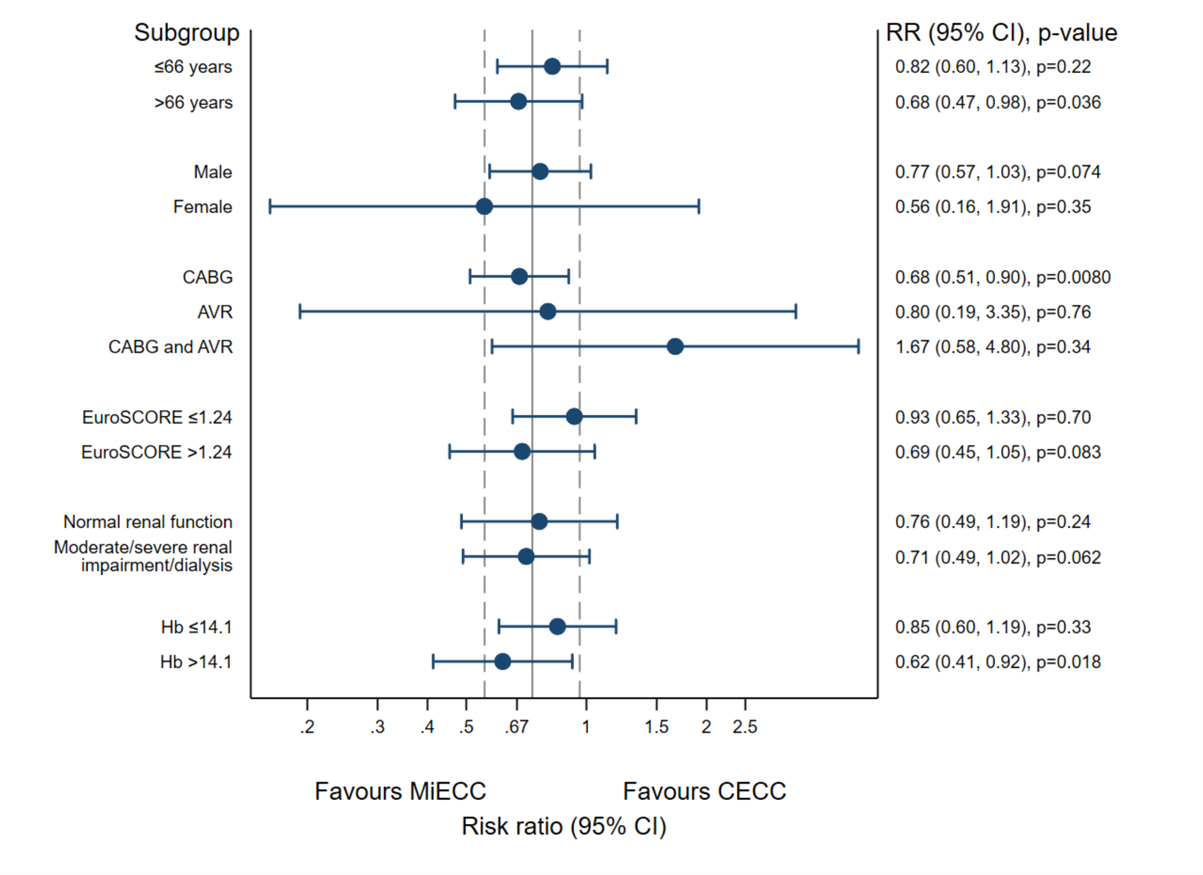

Figure SF1: treatment effects for the six prespecified subgroups. The solid and dashed vertical lines represent the treatment effect and 95% CI from the primary analysis.

AVR – aortic valve replacement; CABG – coronary artery bypass grafting; CECC – conventional extra-corporeal circulation; Hb – haemoglobin; MiECC – minimally invasive extra-corporeal circulation; RR – risk ratio
